# Burkitt Lymphomas Evolve to Escape Dependencies on Epstein-Barr Virus

**DOI:** 10.3389/fcimb.2020.606412

**Published:** 2021-01-11

**Authors:** Rebecca L. Hutcheson, Adityarup Chakravorty, Bill Sugden

**Affiliations:** McArdle Laboratory for Cancer Research, University of Wisconsin–Madison, Madison, WI, United States

**Keywords:** Burkitt lymphoma, B cell, tumor evolution, cellular mutations, Epstein–Barr virus, next-generation sequencing, compensation

## Abstract

Epstein–Barr Virus (EBV) can transform B cells and contributes to the development of Burkitt lymphoma and other cancers. Through decades of study, we now recognize that many of the viral genes required to transform cells are not expressed in EBV-positive Burkitt lymphoma (BL) tumors, likely due to the immune pressure exerted on infected cells. This recognition has led to the hypothesis that the loss of expression of these viral genes must be compensated through some mechanisms. Recent progress in genome-wide mutational analysis of tumors provides a wealth of data about the cellular mutations found in EBV-positive BLs. Here, we review common cellular mutations found in these tumors and consider how they may compensate for the viral genes that are no longer expressed. Understanding these mutations and how they may substitute for EBV’s genes and contribute to lymphomagenesis can serve as a launchpad for more mechanistic studies, which will help us navigate the sea of genomic data available today, and direct the discoveries necessary to improve the treatment of EBV-positive BLs.

## Introduction

Infectious agents cause approximately 2.2 million cases of cancer each year (de Martel et al., 2020), about 15% of all human cancers worldwide (Parkin, 2006; de Martel et al., 2012; Plummer et al., 2016; de Martel et al., 2020). Most of these cancers are caused by tumor viruses. In particular, some cases of Burkitt lymphoma (BL) are caused by Epstein-Barr Virus (EBV), the first human tumor virus to be discovered. In the decades since the discovery of EBV, we have learned that contributions that EBV makes to the formation and maintenance of Burkitt lymphomas are complex. In this review, we consider how Burkitt lymphomas evolve to lose some of their dependencies on this oncogenic virus.

In the 1950s, Denis Burkitt observed a childhood tumor common in Uganda characterized by malignant growths on the jaw and within the abdominal cavity (Burkitt, 1958). Burkitt pursued study of the tumor, recognizing it to be its own clinical entity. He soon appreciated that the large areas of Africa in which the tumor was found overlapped with regions in which rainfall was favorable for holoendemic malaria. Burkitt hypothesized an infectious agent as the cause and collaborated with Anthony Epstein, Yvonne Barr, and Bert Achong, who identified a herpesvirus within tumor cells (Epstein et al., 1964). More than 55 years later, we know that EBV not only causes BL in some populations, but also other cancers of lymphoid and epithelial origin. The World Health Organization classifies BL into three subtypes, endemic BL, sporadic BL, and HIV-associated BL (Swerdlow et al., 2016). All BL are characterized by similar histology, hypermutated immunoglobulin gene sequences, and almost all carry chromosomal translocations in which the c-Myc proto-oncogene is brought under control of one of three immunoglobulin loci, resulting in its constitutive expression and fostering proliferation. While EBV can be found in all three subtypes of BL, each subtype varies in the frequency with which the virus is found. The “high incidence” endemic BL (eBL) found in equatorial Africa and Papua New Guinea is about 95% EBV-positive, whereas in other parts of the world, sporadic BL (sBL) can be anywhere from 20%–80% EBV-positive depending on the geographical region (Magrath, 2012). The endemic and sporadic classification was begun initially to define the two geographical groups of the disease, but more recent analyses of the mutational burden in primary tumors from both eBL and sBL has revealed that EBV presence distinguishes a specific BL phenotype regardless of geographic origin (Kaymaz et al., 2017; Grande et al., 2019).

Though initially controversial, it is now clear that EBV’s presence in Burkitt lymphomas does not merely reflect its being a passenger in these B cell tumors. There are several compelling pieces of evidence supporting EBV’s causal role in the development of BL. First, in a prospective study in Uganda, antibodies against EBV were found to be exceptionally high in children who eventually develop eBL (de-Thé et al., 1978). Second, molecular analysis found that EBV infection precedes the malignant B cell outgrowth (Raab-Traub and Flynn, 1986; Neri et al., 1991). When EBV circularizes its genome on infecting cells, its terminal repeats are joined, with different numbers of repeats characterizing each circularized DNA (Kintner and Sugden, 1979). BL tumor cells harbor multiple EBV genomes, but within individual BL, cells contained EBV DNAs with the same number of terminal repeats, indicating the tumor likely arose clonally following infection with a single virus (Raab-Traub and Flynn, 1986). Third, we have found that only 84% of EBV genomes—which exist as extrachromosomal plasmids in infected cells—in a population are synthesized each S-phase, indicating that if that population of cells is to maintain the virus, EBV must provide a selective advantage to those cells or its DNA will be lost as the cells proliferate (Nanbo et al., 2007). EBV-positive BL cells maintain the viral genome as plasmids over many generations, and thus, EBV must provide a selective advantage to these tumors (Adams and Lindahl, 1975). Consistent with this prediction, for example, when EBV is evicted from EBV-positive BL cell lines, they die by apoptosis (Kennedy et al., 2003; Vereide and Sugden, 2011).

EBV is a unique pathogen in that it not only infects primary B cells but also induces them to proliferate, (a process hereafter called “transformation”). For EBV to transform a cell it infects, the virus must express certain viral genes. These “transforming” viral genes have been experimentally identified under most conditions as being necessary for transformation in cell culture and include EBNA1 (Yates et al., 1985), EBNA2 (Hammerschmidt and Sugden, 1989), EBNA3A and EBNA3C (Tomkinson et al., 1993), and LMP1 (Wang et al., 1985). EBV lacking any one of these genes cannot transform B cells in culture, with the exception of LMP1, which can be substituted for by growing infected cells on a human fibroblast feeder layer (Dirmeier et al., 2005). In addition to these necessary genes, the virus expresses additional genes that increase the efficiency of transforming B cells, namely LMP2, the viral miRNAs, the small, non-coding RNA EBERs, and the transiently expressed, immediate early genes BZLF1 and BRLF1 (reviewed in Young et al., 2016). EBV also encodes circular RNAs that are derived from multiple transcripts and are candidates for regulating viral gene function, at least by serving as sponges for viral miRNAs (reviewed in Ungerleider et al., 2019).

**Figure 1:**
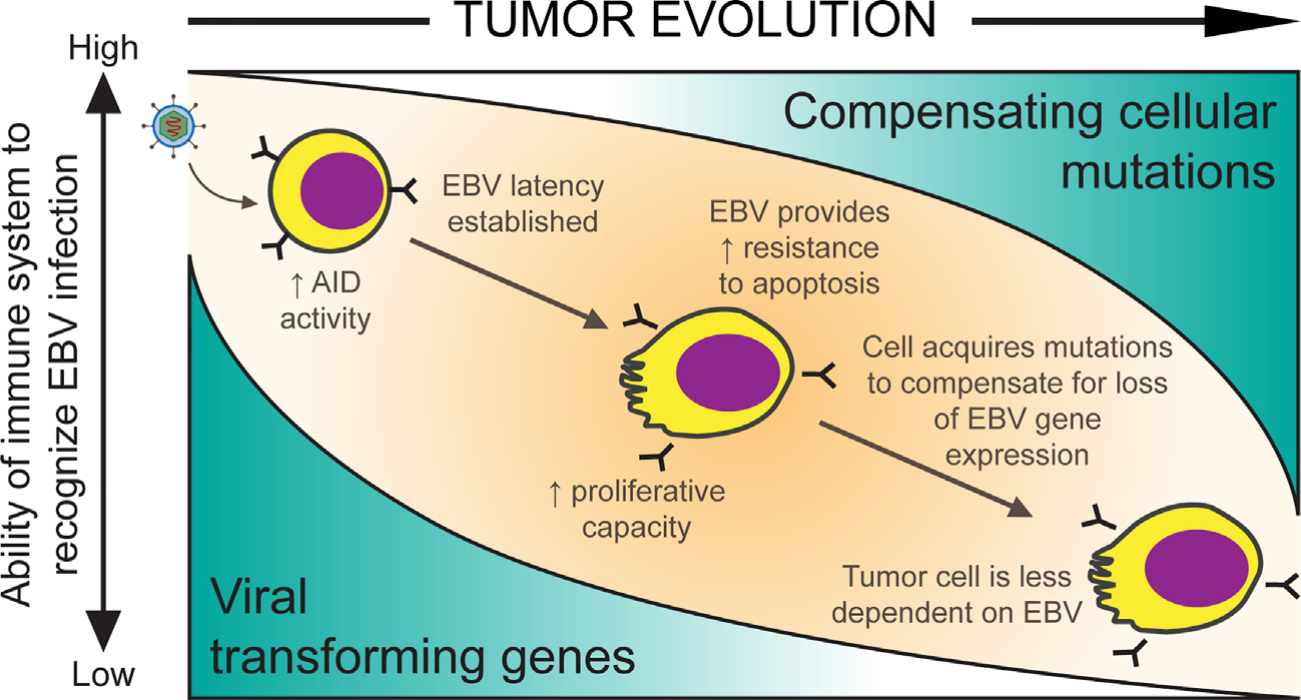
A model for EBV-positive Burkitt lymphomagenesis. Cells transformed with EBV are provided with oncogenic advantages including the ability to proliferate and the ability to avoid apoptosis through the expression of viral transforming genes. However, EBV infected cells are targeted by the host’s immune system. As mutations randomly accumulate in cells, they allow expression of some of EBV’s genes to be turned off if compensated by such cellular mutations and reduce immune-mediated tumor cell killing.

Strikingly, EBV-positive BL tumors often do not express many of these transforming genes including EBNA2, EBNA3A, EBNA3C, LMP1, and LMP2. (Rowe et al., 1986; Niedobitek et al., 1995; Tao et al., 1998). Thus, on the journey from infected B cell to malignancy, EBV-positive tumor cells evolve to reduce viral gene expression to a limited set of viral genes. We propose that cellular mutations are acquired in these tumors that compensate for the lost viral genes (**Figure 1**). Since it is unlikely that EBV drives the occurrence of these cellular mutations, we suspect that as cellular mutations arise by chance, those cells with mutations that provide selective advantages are able to lose the expression of viral genes with similar functions. The loss of viral gene expression in EBV infected cells *in vivo* may be driven by the immune response. This hypothesis is supported by an observation found in another malignancy caused by EBV, post-transplant lymphoproliferative disorder (PTLD), where therapeutically immunosuppressed patients develop a rapid outgrowth of EBV infected cells after organ transplantation. When PTLDs arise quickly (a median of less than 1 year following transplantation) in these immunosuppressed patients, the malignant cells responsible are commonly infected with EBV and express the full set of viral latent proteins. However, in late-onset PTLD, cells express fewer numbers of EBV’s genes (Timms et al., 2003). This observation serves as an example of how EBV-positive cells can evolve over time to express fewer viral genes. In this review, we consider how EBV-transformed B cells evolve as BLs to shut off the expression of some viral genes necessary for transformation and substitute for their functions through cellular mutations.

## Pathways Leading to the Loss of Expression of Viral Genes in Burkitt Lymphoma

Multiple EBV genes are necessary to transform B cells but are no longer expressed in many EBV-positive BL tumor cells. These genes have been found to be downregulated, deleted, or otherwise transcriptionally silenced. The loss of their expression likely reflects the constant pressure of the host’s immune system on viral gene products. While people remain infected for their lives with EBV, the virus typically remains latent in memory B cells (Babcock et al., 1998) and only occasionally is found to be reactivated in the saliva (Niederman et al., 1976). These latently infected memory B cells express few or no viral proteins, so they are unrecognized by the immune response. An observation consistent with this likelihood is that when BL biopsies that express few viral genes are explanted into cell culture, they often re-express additional viral genes, presumably reflecting a lack of immune selection (Rowe et al., 1986; Rowe et al., 1987; Tao et al., 1998). This is also echoed by the earlier onset PTLD in which cells responsible for the disease typically express the full set of EBV latency genes due to the lack of immune surveillance from the host. Consistent with the idea that immune pressure influences EBV gene expression, B cells infected with EBV *in vitro* continue to express the full repertoire of latent viral proteins, known as latency III. In contrast, BL cells usually express a smaller subset of the viral latency genes, known as latency I. Here, we focus on BL specifically, its distinctive pattern of viral gene expression (see **Table 1**) and consider the evidence that supports viral genes being downregulated in these tumors.

**Table 1:** EBV viral gene expression.

Latency Type		Viral mRNAs	Viral noncoding RNAs	Immunogenicity*
**Latency I**	Canonical BL	EBNA1	BART miRNAs EBERs	Limited antigen expression
**Wp Latency**	Wp-restricted BL	EBNA1 EBNA3A, 3B, 3C EBNA-LP (truncated) BHRF1	BART miRNAs EBERs BHRF1 miRNAs	Increased antigen expression
**Latency III**	LCLs	EBNA1 EBNA2 EBNA3A, 3B, 3C EBNA-LP BHRF1 LMP1 LMP2A, 2B	BART miRNAs EBERs BHRF1 miRNAs	Highest antigen expression

*Rowe et al., 1986; Rooney et al., 1986; Rowe et al., 1987.

Annotations in acronym or abbreviated form are: LCL, lymphoblastoid cell line.

Early studies of EBV-positive BL biopsies detected limited viral gene expression, often only EBNA1, among the known transforming genes (Rowe et al., 1987). When the biopsies were grown in cell culture they were found eventually to express the additional transforming genes, EBNA2 and LMP1 (Rowe et al., 1987). In addition to EBNA1, BL biopsies are known to also express viral miRNAs and the EBERs (Niedobitek et al., 1995; Tao et al., 1998). The viral miRNAs and EBERs are thought to be non-immunogenic. EBNA1, on the other hand, apparently avoids immune detection in BL patients. While it is recognized by a CD4^+^ T cell response, this response is inhibited in BL patients (Moormann et al., 2009). Other EBV transcripts have also been detected in some BL tumors but only in a subset of cells (Niedobitek et al., 1995; Xue et al., 2002).

**Figure 2:**
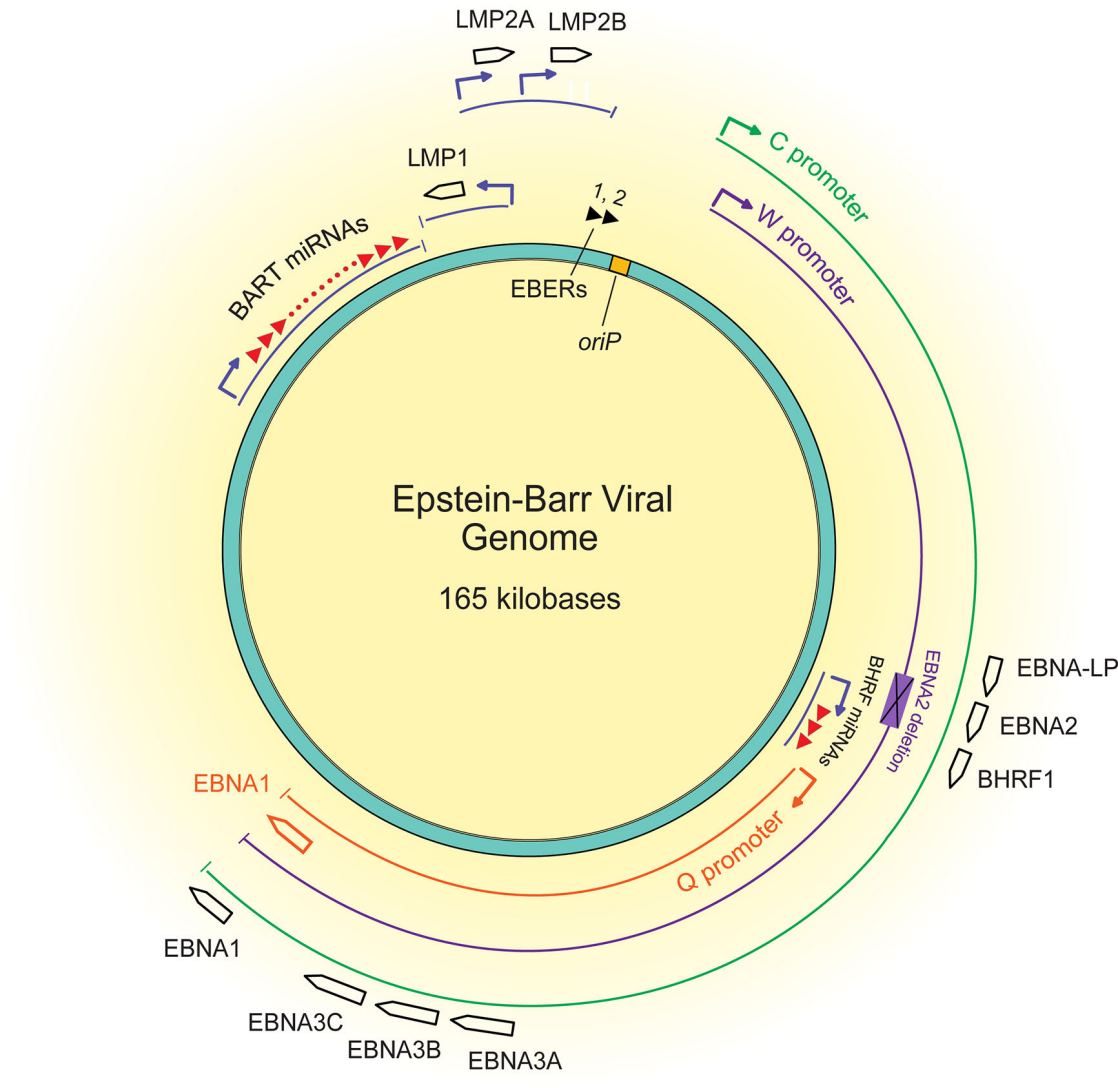
A map of the latent EBV genome. The 165 kilobase pair double-stranded DNA genome exists as a plasmid maintained within the nucleus of infected cells. The origin of plasmid replication (oriP) is shown in yellow. The thin lines represent transcripts, each color representing the promoter that drives their transcription, C (green), W (purple), and Q (orange). The black boxed arrows represent the approximate locations of exons encoding latent proteins. The black triangles at the top represent the highly transcribed non-coding RNA EBERs, and the red triangles represent the two sets of miRNAs.

About 15% of African BLs have deletions of the EBNA2 transforming gene, are termed Wp-restricted BL (Kelly et al., 2002), and as the name implies, express genes from the W promoter (**Figure 2**). This subset of BLs expresses several additional transforming genes, including EBNA3A, EBNA3B, EBNA3C, truncated EBNA-LP, and BHRF1. It is unclear how these EBNA2 deletions occur mechanistically, but they likely reflect a selection against EBNA2 during the evolution of these BLs. Early genetic analyses of EBV’s transforming genes identified EBNA2 as essential for transformation (Hammerschmidt and Sugden, 1989), but CD8^+^ T cells targeting EBNA2 efficiently recognize EBV infected B cells at very early time points post-infection (Brooks et al., 2016), potentially indicating an early selection against EBNA2. EBNA2 was found to suppress expression of IgH, leading to decreased Myc expression in BL cell lines (Jochner et al., 1996). Thus, infected cells that can shut down expression from the C promoter or delete EBNA2 entirely can benefit from higher Myc levels. Although viral transforming genes expressed in Wp-restricted BLs can be immunogenic, they can also provide BLs a selective advantage. For example, EBNA3A and EBNA3C block apoptosis by cooperating to inhibit transcription of the pro-apoptotic gene Bim (*BCL2L11*) (Anderton et al., 2008; Price et al., 2017). Additionally, BHRF1, a viral Bcl2 homologue, can block apoptosis in BL cell lines as can some viral miRNAs (Kelly et al., 2009; Watanabe et al., 2010; Vereide et al., 2014). Evicting EBV from various BL tumor cell lines has shown that these tumor cells rely on EBV for survival depending on the number of viral transforming genes they express (Vereide and Sugden, 2011). These observations indicate that while some BLs escape dependence on some EBV genes, others continue to employ other viral genes to inhibit apoptosis.

**Figure 3:**
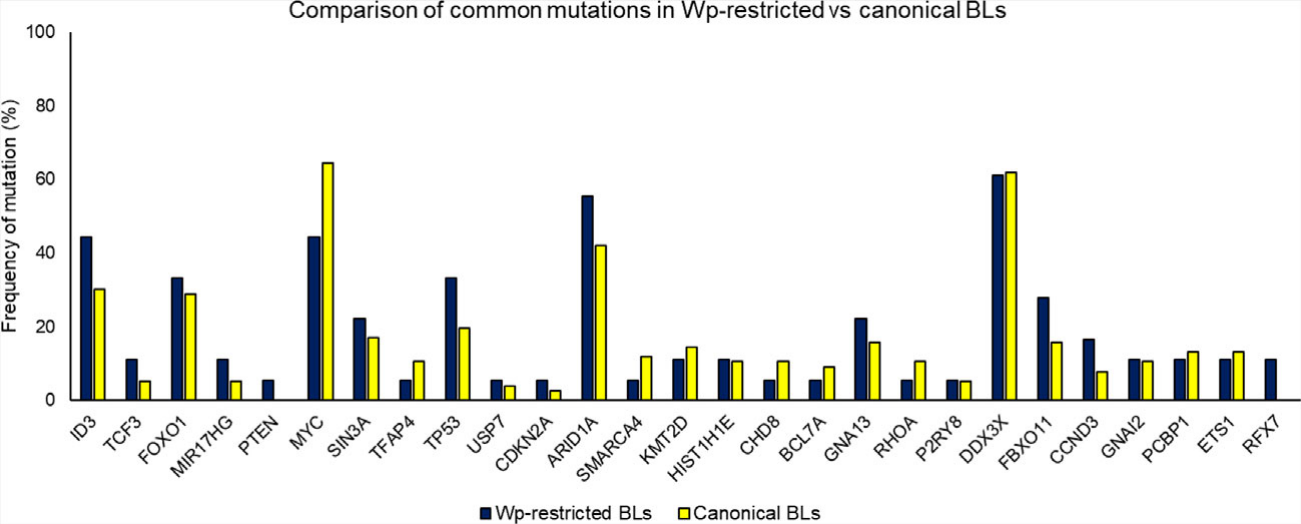
Comparing mutation loads for select genes in Wp-restricted (blue bars) and canonical (yellow bars) Burkitt lymphoma tumors. Data are derived from publicly available datasets (Grande et al., 2019). Tumors were designated EBV-positive based on detectable levels of EBNA1. EBV-positive tumors were categorized as Wp-restricted if EBNA2 expression was absent and EBNA3A/EBNA3C expression detected.

Importantly, the overall mutational landscape in Wp-restricted BLs is not significantly different from that in canonical EBV-positive BLs (**Figure 3**), although there are subtle differences in the cellular expression pattern of Wp-restricted versus canonical BL cell lines. For example, Kelly *et al*. find that Wp-restricted BLs consistently display a downregulation of BCL6 and upregulation of IRF4 and Blimp1 (Kelly et al., 2013). Though our analysis of previously available data found no significant differences in the frequency of mutations in the examined genes, one limitation of interpreting these findings is that there were relatively few samples analyzed. Thus, it is likely that Wp-restricted and canonical BLs result from the expansion of similar progenitor cells, perhaps with subtle variations in mutational patterns reflecting differences in selection pressure due to differences in EBV gene expression.

Yet, the question remains: how does an EBV infected B cell evolve to lose expression of some or all of the viral genes initially required for its transformation? Clearly for Wp-restricted BLs, this loss occurs *via* deletions, at least in part. A second route to this loss is likely transcriptional. EBNA1 is expressed from different viral promoters in most BLs than in B cells transformed *in vitro* (Tao et al., 1998; Altmann et al., 2006). The C promoter drives expression of EBNA1 and the immunogenic EBNA proteins in lymphoblastoid cell lines. Wp-restricted BLs use the W promoter to express multiple transforming genes, and most BLs use the Q promoter from which only EBNA1 is expressed. These changes in viral promoters being used illustrate the evolution of BLs and underlie one mechanism by which BLs downregulate viral genes to escape dependence on them.

The evolution of these EBV infected cells is probably a complex process. For example, it is particularly intriguing to consider the hurdles EBV must overcome for its genomes to acquire mutations and subsequently express them successfully. BL cells maintain multiple copies of viral genomes in a single cell. Mutations arise rarely and surely in single viral genomes such that any one cell would have multiple copies of the wild-type viral gene in the presence of a lone mutant allele. Because EBV genomes are partitioned faithfully 88% of the time (Nanbo et al., 2007), a newly acquired viral mutation is likely to take over in daughter cells only if it provides a robust selective advantage.

In addition to several viral proteins, EBV’s miRNAs also contribute to the survival of BL cells (Vereide et al., 2014), presumably without eliciting an immune response. The levels of these miRNAs differ across EBV-positive BLs (Cai et al., 2006; Xia et al., 2008) and across BL cell lines (Pratt et al., 2009). These different levels may also reflect BLs evolving to become independent of EBV. For example, the EBV-positive BL cell lines Daudi, Mutu I, and Akata can all lose EBV DNA spontaneously in culture. Their EBV-positive parents express fewer viral miRNAs than do other cell lines (Pratt et al., 2009), demonstrating, perhaps, that they have evolved to reduce their dependence on these viral miRNAs.

Though there is much evidence for loss of expression of viral genes in BL, either by promoter switching or deletion events, it is not clear yet how BLs compensate for this loss.

## Factors Contributing to Loss of Expression of Viral Genes in Burkitt Lymphoma

The viral genes whose expressions are often lost in BL tumor cells are not random; BL viral gene expression is similar across tumor biopsies. Though we do not fully understand the mechanisms behind this expression pattern, there are pieces of evidence that provide glimpses into the forces that shape the loss of some viral gene expression. For example, in one study in which B cells were transformed by a derivative of EBV in which EBNA2 functioned conditionally and c-Myc was expressed at high levels constitutively, the cells were found to no longer express LMP1 when EBNA2 was inhibited (Polack et al., 1996), but they continued to proliferate. This study shows that c-Myc, if expressed at high enough levels, can compensate for the loss of both EBNA2 and LMP1. Another set of cellular genes that may compensate for the loss of expression of viral genes in BL belongs to the Notch signaling pathway, which is known to be upregulated in BL (He et al., 2009). Notch-2 signaling *via* Delta-like ligand 1 was shown to inhibit EBNA2-mediated initiation of LMP1 transcription (Rowe et al., 2014). In addition, a high level of expression of introduced intracellular Notch in cells in which EBNA2 functions conditionally inhibited the expression of LMP1 (Gordadze et al., 2001). These observations indicate that increased levels of Notch signaling may compensate for the loss of expression of LMP1.

Some BL cell lines are less dependent on the virus for proliferation and survival than others (Vereide and Sugden, 2011). When EBV was evicted from BL cell lines, those BLs that formerly expressed more viral genes underwent apoptosis more rapidly than those that expressed fewer viral genes. One latency I BL which expressed the fewest viral genes continued to proliferate slowly even in the absence of EBV. Several of these lost viral latency genes are essential to transform B cells, which indicates that there must be some sort of functional compensation when these viral genes are lost. This compensation is likely to be through cellular mutations that potentially can be identified through sequencing of primary BL tumors. In the next section, we discuss common cellular mutations found in BL, mutations specific to EBV-positive BL, and how these mutations might compensate for loss of expression of viral genes.

## Mechanisms for Cellular Compensation for the Loss of Expression of Epstein–Barr Virus Genes in Burkitt Lymphoma

### Regulation of Apoptosis

With the recent increase in availability and affordability of genome-wide sequencing, there is now a plethora of information about the cellular mutational burden of tumors, including BLs. Studies comparing EBV-positive BLs to tumors that lack the virus have confirmed parallel findings in cells in culture. EBV-positive tumors have significantly fewer mutations in genes affecting apoptosis compared to EBV-negative tumors (Grande et al., 2019), supporting findings that EBV inhibits apoptosis in BL cell lines. Indeed, many of EBV’s latency genes target apoptotic pathways, and this was hypothesized early when eviction of the virus from BL cell lines caused death by apoptosis (Kennedy et al., 2003). Later experiments with Wp-restricted BL cell lines found that these tumor cell lines were much more resistant to cell death triggers than either EBV-negative or latency I BL lines (Kelly et al., 2005; Kelly et al., 2006). EBNA3A and EBNA3C—which are expressed in Wp-restricted BLs—were shown to downregulate Bim (Anderton et al., 2008), providing a mechanism for the increased resistance to apoptosis observed in this subset of EBV-positive BL tumors. Wp-restricted BLs can also express BHRF1, a viral gene that interferes with Bim and other pro-apoptotic proteins (PUMA, BID, BAK) to prevent apoptosis in BL cells (Desbien et al., 2009; Fitzsimmons et al., 2018; Fitzsimmons et al., 2020). The finding that BLs that lack EBV contain more cellular mutations in apoptotic genes, including significantly more mutations in *TP53* (Grande et al., 2019), further supports the hypothesis that mutations in cellular genes linked to apoptosis can compensate for the loss of EBV’s gene expression in BLs.

### Mutations in Genes Typically Targeted by Epstein–Barr Virus’ miRNAs

In addition to encoding several viral proteins, EBV encodes at least 40 miRNAs that can target cellular genes. In fact, few viral genes have been found to be targets of EBV’s miRNAs (Pfeffer et al., 2004; Riley et al., 2012; Skalsky et al., 2012; Jung et al., 2014), leading to the hypothesis that they are more likely to target cellular genes by translational inhibition or mRNA degradation. Cellular genes targeted by EBV miRNAs may be yet another avenue by which the loss of expression of EBV genes can be compensated for by mutations in cellular genes. These cellular genes, when appropriately mutated, could provide functions akin to EBV’s transforming genes, and thus may be found to also be mutated in EBV-negative BLs. Much is known about how EBV’s miRNAs manage the host immune response to EBV infection (reviewed in Albanese et al., 2017); here we will focus on confirmed cellular targets of EBV’s miRNAs (reviewed in Kuzembayeva et al., 2014) that when mutated may compensate for viral transforming genes that are downregulated in BL tumors. Collectively, when EBV’s BART miRNAs were expressed in cells induced to lose the virus, their presence protected cells from apoptosis in BL cell lines. Further investigation showed the cluster of BART miRNAs targeted Caspase 3 to inhibit apoptosis in these BL cells (Vereide et al., 2014). The BL cell lines used for these experiments only expressed one viral protein, EBNA1, indicating that inhibition of Caspase 3 by EBV’s miRNAs compensated for the loss of other EBV genes that block apoptosis. Another example of cellular functional compensation is likely through miRNA BART6-3p inhibiting PTEN (Cai et al., 2015). Interestingly, Ambrosio *et al*. found that protein levels of PTEN are significantly lower in EBV-positive BLs compared with EBV-negative BLs (Ambrosio et al., 2014). This finding supports a mechanism in which a viral miRNA inhibits translation of PTEN. This idea is also supported by the finding that there are more *PTEN* mutations identified in EBV-negative BLs than EBV-positive BLs, although the overall frequency with which *PTEN* mutations occur in BL is only 4% (Grande et al., 2019). Still, EBV benefits enormously from encoding miRNAs given that they take up relatively little genomic space, are thought to be non-immunogenic, and clearly regulate cellular genes to support transformation. If as emerging evidence suggests, extracellular vesicles and/or exosomes containing viral miRNAs, EBERs, and other small viral RNAs are functional, they may support lymphomagenesis (reviewed in Zhao et al., 2019). Through the secretion of these vesicles to other cells or through expression of viral miRNAs in EBV infected cells, mutations in the cellular targets of EBV’s miRNAs can overcome the requirement for these miRNAs and reduce BLs dependence on EBV.

### Transcriptional Regulation

Multiple studies show that EBV affects the expression of cellular genes transcriptionally, including through epigenetic mechanisms. For example, Hernandez-Vargas *et al*. found that the presence of the virus was the most significant variable defining variation in DNA methylation in EBV-positive versus EBV-negative BL cell lines (Hernandez-Vargas et al., 2017). The same study also found that two cellular genes commonly mutated in BL, *TCF3* and its negative regulator *ID3*, had different methylation patterns depending on EBV’s presence or absence. In multiple genome-wide analyses of BL primary tumors, mutations in cellular genes involved in epigenetic regulation were significantly more frequent in EBV-positive biopsies (Kaymaz et al., 2017; Grande et al., 2019; Panea et al., 2019). These include *KMT2D*, *HIST1H1E*, *CHD8*, and *BCL7A*, with some additional genes identified that involve epigenetic regulatory pathways including *DNMT1*. Both LMP1 and LMP2A have been found to increase expression of DNA methyltransferases including DMNT1 in carcinomas, leading to inhibition of E-cadherin (*CDH1*) and *PTEN* expression (Tsai et al., 2006; Hino et al., 2009).

EBNA2 and EBNA3A, 3B, and 3C regulate transcription by interacting with cellular transcriptional machinery. EBNA2 was found to bind the members of the SWI/SNF complex involved in enhancer function (Wu et al., 1996; Alver et al., 2017) while all four EBV proteins can associate with the cellular transcription factor, RBPJ, to different extents to foster transcription from partially overlapping but distinct subsets of genes (Wang et al., 2016). This virus-mediated transcriptional regulation must be overcome by alterations in the cellular machinery to allow the loss of expression of EBNA2 and the EBNA3 transforming genes as BLs evolve.

A different mechanism has been uncovered for EBNA1 to contribute to the transcription of cellular genes. Coppotelli *et al*. have emphasized the similarity of EBNA1’s AT-hook domain to High Mobility Group A (HMGA) proteins (Coppotelli et al., 2013). They have found that EBNA1, similarly to HMGA proteins, can aid in de-condensation of chromatin and increase the mobility of histone H1. This role for EBNA1 is presumably mediated by its specific binding to sites in cellular DNA (Dresang et al., 2009). While it is possible to envision mechanisms in which EBNA1’s mimicry of HMGA proteins is abrogated, its role in maintaining EBV genomes as plasmids appears essential.

A recent approach has identified cellular genes that regulate expression of EBV genes and revealed that both DNMT1 and UHRF1 are instrumental in turning off the expression of multiple viral transforming genes expressed from the C, W, and LMP promoters in BL cell lines (Guo et al., 2020). So long as there are cellular functions, wild-type or mutant, that can compensate for the loss of EBV’s transforming genes, DNMT1 and UHRF1 would foster the escape of BLs from immune recognition of viral antigens.

### Activation-Induced Cytidine Deaminase Off-Target Mutations

The surge in genome-wide mutational data across BL tumors is valuable; however, it can be difficult to determine which mutations may be important in the pathogenesis of BL, and which are merely “passenger” mutations. While combing through the hundreds of mutations identified across multiple studies, it has become clear that some of the reported mutations result from off-target modifications by activation-induced cytidine deaminase (AID), where off-target is defined as any non-Ig loci. AID is an enzyme responsible for class-switch recombination and affinity maturation of antibodies by somatic hypermutation (SHM) and is specifically expressed in germinal center B cells (Muramatsu et al., 1999). AID acts by deaminating cytidine residues in DNA, thus introducing U:G mismatches (Di Noia and Neuberger, 2007). If regulated properly, AID functions to increase Ig gene diversification in developing B cells and is primarily restricted to Ig genes. The enzyme preferentially deaminates cytosines located within WRCY/RGYW regions (Dorner et al., 1998); however, these “hotspot motifs” are also found elsewhere across the genome. Studies show that AID has off-target activity for non-Ig loci, resulting in off-target lesions and chromosomal translocations (reviewed in Chandra et al., 2015). Thus, the regulation of this enzyme is critical to maintain genomic integrity.

Because almost all BL tumors contain c-Myc translocations to one of three Ig loci, researchers have hypothesized for years that AID and EBV are somehow intertwined. The evidence for this hypothesis was slowly built over time; for example, Ramiro *et al*. showed that AID is required for Myc/IgH translocations *in vivo* (Ramiro et al., 2004), and later found that AID causes these same translocations in B cells in culture (Ramiro et al., 2006). Studies done using primary B cells infected with EBV showed an increase in AID expression (Epeldegui et al., 2007), leading researchers to probe further for the link between AID and tumorigenesis of BL. The cellular miR-155 suppresses AID expression (Dorsett et al., 2008; Teng et al., 2008), but miR-155 does not appear to be expressed in primary BL tissue (Kluiver et al., 2006). More recently, the EBV gene EBNA3C has been shown to directly induce AID in B cells (Kalchschmidt et al., 2016), and further studies have shown that some common mutations found in BL are likely to be caused by AID and reflect EBV’s dysregulation of AID.

Across studies investigating the mutational spectrum of EBV-positive versus EBV-negative BLs, researchers have found fewer cellular “driver” mutations but more non-coding mutations in EBV-positive BLs (Abate et al., 2015; Grande et al., 2019; Panea et al., 2019). Two of these studies reported that EBV-positive BLs were associated with a higher proportion of AID-associated mutations compared with EBV-negative BLs (Grande et al., 2019; Panea et al., 2019) (see **Table 2** for a list of AID-associated mutations found in EBV-positive BL). In particular, Grande *et al*. found AID recognition sites were mutated at higher than expected rates. These and other AID-associated mutations can be found in certain genes where the DNA contains G-quadruplex (G4)-containing substrates mimicking the mammalian immunoglobulin switch regions (Qiao et al., 2017) along with the known AID recognition motifs. In fact, many genes identified to have a high frequency of GGG in G-rich regions by Qiao *et al*. were found to be mutated in more than 20% of EBV-positive BL tumors. From these data, it appears that EBV-enhanced expression of AID increases the number of somatic mutations found in BL. While EBNA3C, which was found to induce AID in B cells, is not expressed in many EBV-positive BL, other factors may foster expression of AID in BLs. The cellular protein Blimp1 has been found to inhibit AID through multiple mechanisms (Xu et al., 2007). Intriguingly, Blimp1 was found to have decreased expression in latency I BL cell lines compared to BL lines that had reverted to a latency III phenotype (Kelly et al., 2013). Downregulation of Blimp1 in latency I BLs may serve as one mechanism to increase AID activity without the need for expression of EBNA3C, which is typically not expressed in latency I. This observation could indicate that BLs can continue to accumulate AID off-target mutations indirectly through manipulation of other cellular processes.

**Table 2:** Frequency of some AID off-target genes^1^ mutated in EBV-positive BL.

Gene	Frequency (sample size)	Name	Description ^†^	Compensation for EBV ^†^
***BACH2***	97% (n = 39)^2^	BTB Domain And CNC Homolog 2	–	–
*MYC* *	50% (n = 20)^3^ 60% (n = 30)^4^ 61% (n = 94)^5^ 97% (n = 39)^2^	v-myc myelocytomatosis viral oncogene homolog	TF that drives cell-cycle progression and transformation. Translocation of *MYC* to one of three immunoglobulin loci causes constitutive expression.	Mutations in *MYC* could compensate for EBV’s genes that sustain proliferation
***BCL6*** ^a^	56% (n = 39)^2^	BCL6 Transcription Repressor	Transcriptional repressor. In GC B-cells, represses genes that function in differentiation, inflammation, apoptosis and cell cycle control, also autoregulates its transcriptional expression and up-regulates, indirectly, the expression of some genes important for GC reactions, such as AID, through the repression of microRNAs expression, like miR155.	Mutations in *BCL6* could compensate for EBNA3C through its direct interaction with this protein (Pei et al., 2017)
***BTG2***	51% (n = 39)^2^	BTG Anti-Proliferation Factor 2	–	–
***TCL1A***	41% (n = 39)^2^	T Cell Leukemia/Lymphoma 1A	–	–
***ID3***	30% (n = 20)^3^ 33% (n = 30)^4^ 33% (n = 94)^5^ 62% (n = 39)^2^	Inhibitor of DNA binding 3	HLH protein lacking DNA-binding domain. Functions as a negative regulator of *TCF3*, inhibiting transcription. Mutations are inactivating which decrease *TCF3* interaction.	See *TCF3*
***DNMT1***	36% (n = 39)^2^	DNA Methyltransferase 1	Methylates CpG residues. Preferentially methylates hemimethylated DNA.	Mutations in *DMNT1* could compensate for its interaction with LMP1 and LMP2A (Tsai et al., 2006; Hino et al., 2009).
***MCL1***	33% (n = 39)^2^	MCL1 Apoptosis Regulator, BCL2 Family Member	–	–
***ARID1A***	25% (n = 20)^3^ 13% (n = 30)^4^ 45% (n = 94)^5^ 46% (n = 39)^2^	AT rich interactive domain 1A	–	–
***ETS1***	13% (n = 94)^5^ 51% (n = 39)^2^	ETS Proto-Oncogene 1, Transcription Factor	–	–
***DTX1***	31% (n = 39)^2^	Deltex E3 Ubiquitin Ligase 1	–	–
***PVT1*** ^b^	28% (n = 39)^2^	Pvt1 Oncogene	–	–
***GNA13***	17% (n = 30)^4^ 17% (n = 94)^5^ 46% (n = 39)^2^	Guanine nucleotide binding protein, alpha 13	–	–
***BCL7A***	13% (n = 30)^4^ 9% (n = 94)^5^ 56% (n = 39)^2^	B-cell CLL/lymphoma 7A	–	–
***ZFP36L1***	26% (n = 39)^2^	ZFP36 Ring Finger Protein Like 1	–	–
***TMSB4X***	23% (n = 39)^2^	Thymosin Beta 4 X-Linked	–	–
***CXCR4***	21% (n = 39)^2^	C-X-C Motif Chemokine Receptor 4	–	–
***TFAP4***	17% (n = 30)^4^ 10% (n = 94)^5^ 18% (n = 39)^2^	Transcription Factor AP-4 (activating enhancer binding protein 4)	–	–
***TCF3***	15% (n = 20)^3^ 10% (n = 30)^4^ 6% (n = 94)^5^ 28% (n = 39)^2^	Transcription Factor 3 (E2A immunoglobulin enhancer binding factors E12/E47)	TF that plays a critical role in lymphocyte development. It has also been shown to directly enhance Notch signaling targets. Mutations lead to gain of function.	Gain of function mutations in *TCF3* could compensate for EBNA2, 3A, 3B, 3C and/or LMP2A (Robertson et al., 1996; Gordadze et al., 2001; Portis and Longnecker, 2004; Wang et al., 2016).
***PTEN***	1% (n = 9)^5^	Phosphatase And Tensin Homolog	Functions as a tumor suppressor by negatively regulating AKT/PKB signaling pathway.	Mutations in *PTEN* could compensate for the inhibition of PTEN by EBV’s miRNA BART6-3p (Cai et al., 2015)

*This frequency does not include MYC translocations.

a Identified dependency factor for BL cell line P3HR1 (Ma et al., 2017).

b Significantly more frequently mutated in EBV-positive BL (Grande et al., 2019).

^†^The “Description” and “Compensation for EBV” columns in genes marked with (-) have been intentionally left blank since there is not enough information to interpret their contributions to these tumors.

Annotations in acronym or abbreviated form are: TF, transcription factor; AID, activation-induced cytidine deaminase; HLH, helix-loop-helix; GC, germinal center.

1. Álvarez-Prado et al., 2018; 2. Panea et al., 2019; 3. Abate et al., 2015; 4. Kaymaz et al., 2017; 5. Grande et al., 2019.

Although many of these mutations induced by AID may not contribute to the pathogenesis of BL, some mutations clearly do. *MYC* has long been identified as a target of AID, and the resulting translocation drives the increased proliferation of these tumors. Additionally, genes encoding the transcription factor TCF3 and its negative regulator ID3 are targets of aberrant SHM (Rohde et al., 2017; Álvarez-Prado et al., 2018). Both have been recurrently identified as playing a role in BL pathogenesis, although these mutations have been found significantly more frequently in EBV-negative BL (Grande et al., 2019). Molecular analyses of tumors by Schmitz *et al*. found that BLs are dependent on TCF3 in part because it enhances pro-survival PI3K signaling (Schmitz et al., 2012). The activation of PI3K signaling is a hallmark of BLs (Sander et al., 2012). This upregulation of TCF3 might be seen more frequently in EBV-negative tumors because Notch signaling is mimicked by EBNA2, 3A, 3B, and 3C in EBV-positive tumor cells (Henkel et al., 1994; Waltzer et al., 1994; Zimber-Strobl et al., 1994; Grossman et al., 1994; Robertson et al., 1996; Zhao et al., 1996; Hsieh et al., 1997; Gordadze et al., 2001; Wang et al., 2016). Given that the mutations in *TCF3* are usually gain of function (Schmitz et al., 2012), these mutations could promote cells becoming independent of EBV. Another potential cellular compensation through gain of function *TCF3* mutations is the loss of expression of LMP2A, which is known to activate the PI3K pathway to mediate B cell survival (Portis and Longnecker, 2004). TCF3 increases expression of the BCR, leading to increased PI3K signaling (Schmitz et al., 2012). Thus, a mutation that removes the need to stimulate the PI3K pathway might allow for the loss of expression of LMP2A. It is even possible that these mutations are found in EBV-negative BLs because they lost their dependence on EBV—and therefore no longer maintain EBV DNAs—following their acquisition of these mutations in *TCF3* along with other compensatory cellular mutations.

*BCL6* mutations are another example of an AID off-target site that has significance for the tumorigenesis of BL. BCL6 is a transcriptional repressor required for germinal center formation and antibody affinity maturation. It has been linked to upregulating AID expression because it represses miR-155, which suppresses AID (Basso et al., 2012). BCL6 expression has been found to be increased in latency I BL cell lines compared to latency III lines (Kelly et al., 2013). In accordance with this observation, BCL6 has also been found to mediate degradation of EBNA3C (Pei et al., 2017). Perhaps in latency type I BL cells which do not express EBNA3C and thus are no longer dependent on it, the elevated expression of BCL6 is compensating for the loss of EBNA3C. The loss of *BCL6* from a BL cell line was found to inhibit growth or survival (Ma et al., 2017), indicating that BCL6 can provide BLs a selective advantage.

### Burkitt Lymphoma Cellular Compensatory Mutations

While AID does give rise to many mutations in BL, some commonly found mutations are not known to be off targets of AID activity. These mutations also have the potential to compensate for EBV’s transforming genes allowing their expression to be lost in BL tumors. **Table 3** includes a list of some of the cellular mutations frequently found in EBV-positive BL tumors. The mutations listed have been selected based on their frequency, their known phenotypes making them likely to compensate for viral gene functions, and their potential effects on protein function. We have chosen to highlight these specific mutations, yet the complexity in trying to understand all mutations found in BL tumors becomes apparent when considering information that is lacking. For example, many studies do not report whether the mutations identified in BL tumors affect one or both alleles or how these mutations affect levels of gene expression for many of the mutations reported (Schmitz et al., 2012; Abate et al., 2015; Kaymaz et al., 2017; Panea et al., 2019; Grande et al., 2019). Without examining individual mutations in single tumors, examining functional consequences of that mutation, and considering how that mutation affects other mutations, it is difficult to attribute mechanistic significance to mutations in single genes. Stringently examining each mutation reported across genome-wide mutational studies is beyond the scope of this review.

**Table 3:** Frequency of some mutated genes in EBV-positive BL.

Gene	Frequency (sample size)	Name	Description	Compensation for EBV
***IGLL5*** ^a^	97% (n = 39)^1^	Immunoglobulin lambda-like polypeptide 5	Located within the immunoglobulin lambda locus but does not require somatic rearrangement for expression.	Mutations in *IGLL5* could compensate since *IGLL5* is regulated by EBV (Zhou et al., 2015)
***DDX3X***	35% (n = 20)^2^ 40% (n = 30)^3^ 62% (n = 94)^4^ 82% (n = 39)^1^	DEAD (Asp–Glu–Ala–Asp) box polypeptide 3, X-linked	ATP-dependent RNA helicase.	Unknown
***IKZF3***	44% (n = 39)^1^	IKAROS Family Zinc Finger 3	TF that plays an essential role in regulation of B-cell differentiation, proliferation, and maturation to an effector state.	Mutations in *IKZF3* could compensate since *IKZF3* is regulated by EBV (Zhou et al., 2015)
***FOXO1***	7% (n = 30)^3^ 30% (n = 94)^4^ 56% (n = 39)^1^	Forkhead box O1	Key TF regulated by the PI3K/AKT pathway. Loss of function mutations may have a role in cell growth or escape from apoptosis.	Unknown
***SIN3A***	18% (n = 94)^4^ 41% (n = 39)^1^	SIN3 transcription regulator family member A	Acts as a transcriptional repressor. Corepressor for REST. Interacts with MXI1 to repress *MYC* responsive genes and antagonize *MYC* oncogenic activities.	Mutations in *SIN3A* could compensate for EBNA3C through interaction with Sin3A (Jiang et al., 2014)
***S1PR2***	28% (n = 39)^1^	Sphingosine-1-Phosphate Receptor 2	Receptor for the lysosphingolipid sphingosine 1-phosphate (S1P). S1P elicits diverse physiological effects on most types of cells and tissues.	Mutations in *S1PR2* could compensate for LMP1 which regulates S1PR2 (Vockerodt et al., 2019)
***FBXO11***	13% (n = 30)^3^ 18% (n = 94)^4^ 36% (n = 39)^1^	F-box protein 11	Substrate recognition component of a SCF (SKP1-CUL1-F-box protein) E3 ubiquitin-protein ligase complex. Major target is *BCL6*. Loss of function mutations.	Mutations in *FBXO11* could compensate for EBNA3C through *BCL6* interaction (Pei et al., 2017)
***CREBBP***	21% (n = 39)^1^	CREB Binding Protein	Acetylates histones, giving a specific tag for transcriptional activation. Also acetylates non-histone proteins, including DDX21, FBL, IRF2, MAFG, NCOA3, POLR1E/PAF53, and FOXO1.	Mutations in *CREBBP* could compensate for EBNA2 through direct interaction with CBP to activate the LMP1 promoter (L. Wang et al., 2000)
***TP53*** ^b^	15% (n = 20)^2^ 17% (n = 30)^3^ 22% (n = 94)^4^ 33% (n = 12)^5^ 15% (n = 39)^1^	Tumor protein p53	Encodes p53, a tumor suppressor that binds to DNA and regulates gene expression. Can activate DNA repair proteins when DNA is damaged and arrest the cell cycle at the G1/S checkpoint, or initiate apoptosis if DNA damage is irreversible. Mutations are loss of function.	Mutations in *TP53* could compensate for EBV’s genes that block apoptosis
***IRF8*** ^a^	18% (n = 39)^1^	Interferon Regulatory Factor 8	Plays a role as a transcriptional activator or repressor. Plays a negative regulatory role in immune cells.	Mutations in *IRF8* could compensate for EBNA3C through its interaction with IRF8 (Banerjee et al., 2013)
***CCND3*** ^a^ ^b^	5% (n = 20)^2^ 13% (n = 30)^3^ 10% (n = 94)^4^ 31% (n = 39)^1^	Cyclin D3	A regulator of progression through G1 phase during cell cycle. Loss of C terminal domain leads to constitutive activation.	Mutations in *CCND3* could compensate for unregulated cell cycle progression
***RHOA*** ^a^	20% (n = 20)^2^ 13% (n = 30)^3^ 10% (n = 94)^4^ 15% (n = 39)^1^	Ras homolog family member A	Small GTpase protein in the Rho family. Regulation of signal transduction between membrane receptors and focal adhesion molecules. Likely loss of function mutations.	Unknown
***USP7*** ^b^	4% (n = 94)^4^	Ubiquitin Specific Peptidase 7	USP7 deubiquitinates target proteins such as p53 and regulates their activities by counteracting opposing ubiquitin ligase activity.	Mutations in *USP7* could compensate for EBNA1 through its interaction (Holowaty and Frappier, 2004)
**Mutations involved in epigenetic regulation** ^b^				
***HIST1H2BK***	49% (n = 39)^1^	Histone H2B		Unknown
***HIST1H3H***	44% (n = 39)^1^	Histone H3.1		Unknown
***HIST1H1E***	11% (n = 94)^4^ 64% (n = 39)^1^	Histone H1.4		Unknown
***HIST1H1C***	28% (n = 39)^1^	Histone H1.2		Unknown
***CHD8***	10% (n = 94)^4^ 26% (n = 39)^1^	Chromodomain Helicase DNA Binding Protein 8	DNA helicase that acts as a chromatin remodeling factor and regulates transcription.	Unknown
***KMT2D*** ^a^	14% (n = 94)^4^ 5% (n = 39)^1^	Histone-lysine N-methyltransferase 2D	Colocalizes with TFs on transcriptional enhancers. Plays critical roles in regulating cell fate transition, metabolism, and tumor suppression.	Unknown

a Identified dependency factor for BL cell line P3HR1 (Ma et al., 2017).

b Significantly more frequently mutated in EBV-positive BL (Grande et al., 2019).

c Significantly more frequently mutated in EBV-negative BL (Grande et al., 2019).

Annotations in acronym or abbreviated form are: TF, transcription factor.

1. Panea et al., 2019; 2. Abate et al., 2015; 3. Kaymaz et al., 2017; 4. Grande et al., 2019; 5. Giulino-Roth et al., 2012.

One challenge in identifying “driver” versus “passenger” mutations in BLs is connecting the mutations to functional effects. Of the common mutations listed in **Table 3**, many were also identified as dependency factors of a BL cell line, P3HR1, using a CRISPR knockout screen (Ma et al., 2017). Deletions in these genes led to decreases in growth or survival of this BL cell line. The examined P3HR1 cell line expresses only EBNA1 among EBV’s latent proteins (an unexpected finding given that other P3HR1 cell lines are typically Wp-restricted), which implies that required functions of the other transforming genes have already been compensated by cellular mutations. The mutated genes found as dependency factors include *IGLL5*, *IRF8*, *CCND3*, *RHOA*, *KMT2D*, and *BCL6*. Of these identified candidates, *IGLL5* (along with another frequently mutated gene, *IKZF3*) is regulated by EBV (Zhou et al., 2015). It is certain that some of the cellular genes regulated by EBV can foster B cell transformation. In EBV-negative cells, mutations in *IGLL5* that increase its activity could compensate for the loss of its typical upregulation by EBV. The CRISPR knockout screen also identified *IRF8*, a transcription factor that is thought to function as a regulator of apoptosis and potentially acts as a tumor suppressor. IRF8 is the only known transcription factor shown to be involved in *BCL6* transcriptional induction (Lee et al., 2006). EBNA3C is thought to regulate IRF8 indirectly through its stabilization of IRF4, which leads to the downregulation of IRF8 by enhancing its proteasome-mediated degradation (Banerjee et al., 2013). In the context of the development of BL, an acquired activating mutation in *IRF8* may compensate for the loss of EBNA3C in tumor cells.

Other mutations identified across mutational analyses are in cellular proteins known to interact with EBV latency proteins. For example, SIN3A is a transcriptional repressor that was found to be recruited by EBNA3C to BATF/IRF4 or SPI1/IRF4 composite sites to repress *CDKN2A* transcription (Jiang et al., 2014). *CDKN2A* codes for both p16 and p14arf, which activate the p53 and pRB pathways, respectively. Loss of EBNA3C expression from an EBV-positive BL would therefore likely require compensatory inhibitions of the p53 and pRB. Mutations in another gene, *FBXO11*, could also be linked to EBNA3C function. *FBXO11* mutations are thought to be loss of function mutations (Duan et al., 2012). One of the major targets of FBXO11-mediated degradation is BCL6 (Duan et al., 2012), which targets EBNA3C for degradation (Pei et al., 2017). The increased expression of BCL6 can provide a growth advantage to BLs. Similarly, mutations in the gene *S1PR2*, which encodes the receptor for the lysosphingolipid sphingosine 1-phosphate, could promote cell proliferation and suppress apoptosis. S1PR2 was shown to be regulated by LMP1 in diffuse-large B cell lymphoma (Vockerodt et al., 2019). In turn, LMP1 transcription is regulated by EBNA2, which was shown to interact with the co-activator CBP (CREB binding protein), encoded by the gene *CREBBP* (Wang et al., 2000). *CREBBP* mutations were detected in more than 20% of BLs.

Other genes frequently mutated in BLs and playing a role in proliferation include *CCND3*, which was significantly more frequently mutated in EBV-negative BL (Grande et al., 2019). This gene encodes cyclin D3, which regulates cell cycle progression through G1. Mutations identified across studies found truncating lesions, and it is understood that the loss of the C terminal domain of *CCND3* leads to constitutive activation (Schmitz et al., 2012). Unregulated cell cycle progression might compensate for EBV’s contributions to BL proliferation were the virus to be lost from BL cells. These mutations found frequently in EBV-positive BL all serve as examples of genes that when mutated could compensate for the loss of EBV’s transforming genes and provide a selective advantage to the cells containing these mutations.

## Concluding Remarks

We hypothesize that EBV-positive tumor cells must have acquired cellular mutations to compensate for the loss of expression of viral genes. This hypothesis leaves multiple questions unanswered but is a starting point for investigating these commonly found mutations in EBV-positive versus negative BLs. One controversial question is: Are some tumors found to be EBV-negative formerly EBV-positive having evolved to be independent of the virus? It has been observed that some BLs spontaneously lose EBV in culture (Akata, MutuI, and Daudi) (Shimizu et al., 1994; Kitagawa et al., 2000; Nanbo et al., 2002). In addition, Grande *et al*. identify *USP7* as being mutated in BL, although relatively rarely. *USP7* encodes a deubiquitinase that counteracts ubiquitination and degradation of proteins such as p53 (Li et al., 2002) and has the mutational pattern of a tumor suppressor in BL. The interaction between p53 and USP7 can be disrupted by EBNA1 (Holowaty and Frappier, 2004). Interestingly, the mutations found in *USP7* were more common in EBV-negative tumors, which obviously do not express EBNA1. *USP7* may serve as one example of a gene that when mutated compensates for EBNA1 and may lead to tumors that have lost EBV genomes, and therefore also lost the expression of EBV latency proteins entirely.

Another question to consider is whether the detection of EBV in tumors is inaccurate. The standard method for assaying for EBV in tumors is through the detection of the EBERs. However, analyses of biopsies have found multiple cases of BL that were EBER-negative and harbored partial viral miRNAs and some genomes (Mundo et al., 2017; Mundo et al., 2020). This hypothesis and the findings consistent with it could indicate that the contribution of EBV to the pathogenesis of BL is greater than previously thought. One technical concern with this notion is that biopsies are typically contaminated with cells other than those of tumor origin, making it difficult to discern if partial or few viral genomes reflect few EBV-positive cells either in the tumor or in contaminating, non-tumor cells. The most compelling evidence for the entire loss of EBV in tumors would be detection of an EBV-positive BL case that subsequently evolves to lose the virus.

We favor the hypothesis that EBV-positive tumors evolve to become less dependent on EBV illustrating the ever-changing selective pressures favoring tumor growth. The surge in data for genome-wide mutational burden in these tumors has tremendous value; however, as more sequencing data becomes available for EBV-associated BL and other EBV-positive malignancies, it will be important to attribute mechanisms to the identified mutations so we might further understand these EBV-associated cancers and their evolution to treat them more effectively.

## Author Contributions

RLH wrote the manuscript with contributions from AC and BS. All authors made a substantial and intellectual contribution to the work, contributed to manuscript revision, read, and approved it for publication. All authors contributed to the article and approved the submitted version.

## Funding

This work was supported by a National Institutes of Health grant (P01 CA022443). RLH was supported by a Cancer Biology Pre-Doctoral Training Grant (T32 CA009135). AC was supported by an NRSA award (T32 A1078985).

## Conflict of Interest

The authors declare that the research was conducted in the absence of any commercial or financial relationships that could be construed as a potential conflict of interest.
